# Global synergies and trade-offs between multiple dimensions of biodiversity and ecosystem services

**DOI:** 10.1038/s41598-019-41342-7

**Published:** 2019-04-04

**Authors:** Marco Girardello, Andrea Santangeli, Emiliano Mori, Anna Chapman, Simone Fattorini, Robin Naidoo, Sandro Bertolino, Jens-Christian Svenning

**Affiliations:** 1 Section for Ecoinformatics and Biodiversity, Department of Bioscience, Aarhus University, Aarhus, Denmark; 2 The Helsinki Lab of Ornithology, Finnish Museum of Natural History, University of Helsinki, Helsinki, Finland; 3 University of Turin, Department of Agronomy, Forestry and Food Sciences, Entomology and Zoology, Turin, Italy; 4 Department of Biology, National and Kapodistrian University of Athens, Athens, Greece; 5 Department of Life, Health and Environmental Sciences, University of L’Aquila, 67100 L’Aquila, Italy; 6 Conservation Science Program, World Wildlife Fund, Washington, DC 20037 USA; 7 Department of Life Sciences and Systems Biology, University of Turin, Via Accademia Albertina, 26-13, 10123 Torino, Italy; 8 Center for Biodiversity Dynamics in a Changing World (BIOCHANGE), Department of Bioscience, Aarhus University, Ny Munkegade 114, DK-8000 Aarhus C, Denmark

## Abstract

Ensuring the persistence of biodiversity and ecosystem services represents a global challenge that need to be addressed with high urgency. Global priority areas can only be identified by means of an integrated prioritization approach that would not only preserve species numbers and ecosystem services, but also the evolutionary and functional components of diversity. In this study we combine global datasets on the distribution of mammals and birds with species traits and phylogenetic data and we identify conservation priorities for taxonomic, functional and phylogenetic diversity, as well as for three ecosystem services, including potential for carbon sequestration, pollination potential and groundwater recharge. We show that, when priority areas are identified based only on individual, e.g. functional diversity, or any combination of the three biodiversity components, these areas do not allow a sufficient protection of the three ecosystem services. However, an integrated approach whereby prioritization is based on all biodiversity components and ecosystem services would allow to identify areas that maximize protection of all ecosystem services with a minimal loss in biodiversity coverage. Our results highlight the need for an integrated conservation planning framework in order to optimally allocate resources and achieve the long-term preservation of the multiple dimensions of biodiversity and ecosystems services.

## Introduction

One of the main targets of the Convention on Biological Diversity is the protection of at least 17% of the terrestrial land areas of particular importance for biodiversity and ecosystem services (Aichi target 11)^1^. Therefore, identifying areas that support high levels of biodiversity and ecosystem services is of crucial importance for the efficient implementation of international policy targets^2,3^. Protection of those important areas would also help preserving the quality of ecosystems that support human-wellbeing, thereby also contributing to achieve the targets of the United Nations Sustainable Development Goals (www.undp.org). In practice, a large overlap between areas protected for biodiversity and ecosystem services would indicate win-win solutions^4,5^. Under this scenario, policies introduced to safeguard biodiversity could simultaneously benefit ecosystem services fundamental for human wellbeing. Conversely, low spatial overlap between areas important for biodiversity and ecosystem services would underpin the emergence of difficult trade-offs^6^. Under this latter scenario specific conservation strategies can be created to ensure the provision of necessary ecosystem services. However, if attention is prominently focused on ecosystem services, there is a risk that limited conservation resources will be diverted away from protecting biodiversity.

The identification of important areas for biodiversity and ecosystem services has typically relied on biodiversity metrics restricted to species numbers i.e. taxonomic diversity (TD)^7^. However, the current consensus is that biodiversity is much more than the simple sum of the species in a given locality. Thus, biodiversity as a whole may be best understood and conserved if other components, namely the phylogenetic and functional diversity, are considered^8^. These components are crucial in conservation planning as they may indicate areas where evolutionary unique species, or species with irreplaceable ecological roles within an ecosystem, can be found^9^. It is widely accepted that functional^10^ and phylogenetic^11^ relationships among taxa are key ecological and evolutionary determinants of biodiversity. Therefore, the use of alternative biodiversity metrics has been argued^12^. While, functional (FD) and phylogenetic (PD) diversity often show a positive relationship with ecosystem services, simple overlap exercises have indicated a lack of spatial concordance between biodiversity and ecosystem services^13^.

Previous large-scale conservation planning assessments typically used taxonomic diversity metrics to quantify synergies and trade-offs between biodiversity and selected ecosystem services^5,14^. These include analyses aimed at examining the spatial overlap between taxonomic diversity and carbon, or a bundle of ecosystem services^15^. However, an integrated prioritization between multiple components of biodiversity and different ecosystem services is currently lacking. In this study we employ spatial prioritisation tools to quantify synergies and conflicts between TD, FD and PD of two vertebrate groups as well as for three ecosystem services (namely carbon storage, pollination and water provision services). We combine distributional, trait and phylogenetic data at the global level. We first quantify the extent of spatial overlap between biodiversity and ecosystem service components from a scenario whereby priority areas are identified using in turn each single biodiversity component. Next, we repeated the above analyses but based on an integrated scenario whereby priority areas are identified using the three biodiversity components combined with the three ecosystem services. The latter would represent a holistic approach whereby the protection of priority areas for biodiversity persistence and selected ecosystem service provision is optimized.

## Results

**Figure 1:**
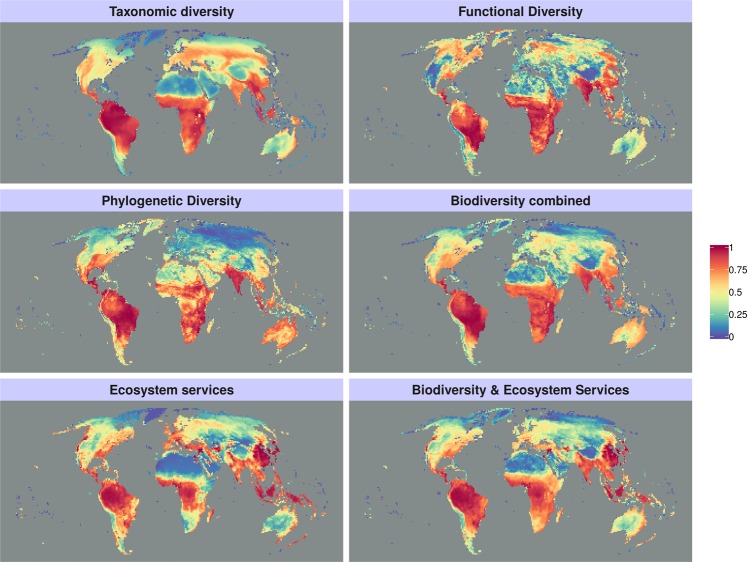
Priority ranking maps showing areas that would be most suitable for the conservation of biodiversity and ecosystem services. These include separate maps showing priorities for taxonomic diversity, functional diversity and phylogenetic diversity, as well as a map showing priorities for all the three diversity components combined. Priority areas for all selected ecosystem services (i.e. carbon biomass, water recharge, pollination services and livestock production on grassland) are also shown, as well as priority areas based on all three biodiversity components as well as ecosystem services combined.

Globally the highest priorities for each of the three biodiversity components (taxonomic, phylogenetic and functional) are largely concentrated in the Global South (Fig. 1). Moreover, in this region priorities identified based on the three different biodiversity components are broadly congruent, mainly covering large areas of South America, Sub-Saharan Africa, South-East Asia and the Indian subcontinent (Fig. 1). Conversely, priorities in the Northern Hemisphere differ markedly between the three biodiversity components, with areas at very high latitudes, such as Canada and Alaska, resulting of relatively high priority for functional diversity only (Fig. 1). As a result, when priorities are identified based on all the three biodiversity components combined, large regions consistent with those mentioned above and based on the single biodiversity component scenarios appear as important for conservation in the Global South. However, the same scenario based on all biodiversity components also identifies as relatively high priority large regions covering the Nearctic, from areas in Greenland to areas in Central America. Several of these latter areas, particularly those at very high latitudes, are not identified as high priority for conservation when only the taxonomic diversity component is considered (Fig. 1). Priorities for all the ecosystem services combined (Fig. 1) largely locate in tropical forest areas, where below and above-ground carbon biomass and groundwater recharge are highest (Fig. S2). Conversely, priorities for all ecosystem services at medium to high latitudes largely concentrate in agricultural areas (e.g. the Mediterranean basin) where services such as pollination are highest, as well as temperate and boreal forest areas high in carbon biomass and water recharge. Finally, global priorities identified based on all three biodiversity components and the ecosystem services tend to highlight areas identified as important for both biodiversity components combined and for the ecosystem services combined by the previous scenarios (Fig. 1). These areas largely concentrate in tropical forests of Central and South America (e.g. Amazon basin), West and Central Africa (e.g. Congo Basin) as well as South and South-east Asia (Fig. 1).

**Figure 2:**
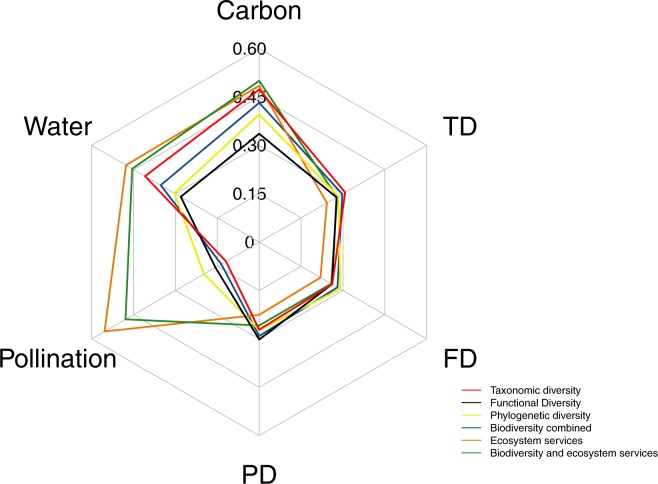
Synergies and trade-offs for biodiversity and ecosystem services. Proportion of each feature retained in the 17% top fraction of the landscape. For biodiversity the proportion is a mean percentage between birds and mammals.

When the outcomes of the alternative prioritization scenarios are quantitatively compared with reference to the 17% top fraction of the landscape being protected (following Aichi Target 11), clear synergies and trade-offs emerge (Fig. 2). These comparisons highlight that when global priorities for conservation are identified with exclusive focus on the biodiversity components, alone or in combination, some of the ecosystem services considered here, such as pollination, are not well represented and would potentially remain largely unprotected (Figs 2, S3). This underscores a trade-off between biodiversity conservation and the preservation of pollination services. Meanwhile, prioritizing only for biodiversity resulted in a high coverage of important areas for carbon biomass and to a lower degree for groundwater recharge, underscoring potential synergies among these different components (Figs 2, S3).

Prioritizing for ecosystem services and biodiversity together would instead result in a marginal loss in biodiversity coverage, but would yield high gains in terms of ecosystem services protection compared to the biodiversity only scenarios (Figs 2, S3). For example, the coverage of priority areas for pollination increase threefold when priorities are identified by considering ecosystem services and biodiversity compared to when only biodiversity is considered (Fig. 2). Ultimately, the most efficient conservation strategy appears to be represented by a combined prioritization of biodiversity and ecosystem services. This solution identifies large global synergies for conserving biodiversity and ecosystem services.

**Figure 3:**
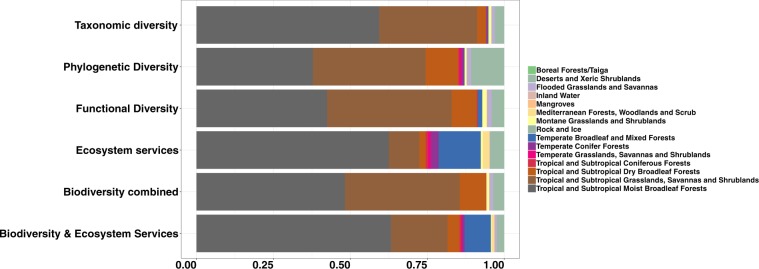
Distribution of the 17% top fraction of the landscape across biomes (the different colors in the bars) as derived from each of the six prioritization scenarios considered (each bar as named on the Y axis).

A biome-level examination of the top 17% fraction of the landscape indicated that for the scenarios considering biodiversity only, priorities largely cover tropical and subtropical broad leaf forests and tropical and subtropical grasslands, savannas and shrublands (Fig. 3). When prioritizing for ecosystem services alone or ecosystem services and biodiversity, the top priority areas still largely represented by tropical and subtropical broad leaf forests and to a lower degree tropical and subtropical grasslands, savannas and shrublands. However, under this scenario other biomes, such as temperate broadleaf and mixed forests and Mediterranean forests are more represented within the top priorities compared to the biodiversity based scenario (Fig. 3).

## Discussion

In this study, we identify potential synergies and trade-offs between conserving the different components of biodiversity and selected ecosystem services at the global level. We show that some important trade-offs appear when aiming to achieve biodiversity conservation and the preservation of ecosystem services, such as pollination. However, we also identify widespread synergies when both biodiversity and ecosystem services are considered for identifying priority areas for protection. Overall, the findings indicate that a joint integration of biodiversity components and ecosystem services allows achieving a much greater preservation of the ecosystem services at a minimal loss in biodiversity protection compared to a scenario where only biodiversity is considered. This underscores the importance of considering the joint contribution of the different dimensions of biodiversity as well as ecosystem services for effectively achieving international conservation policy goals.

While several previous studies have explored the link between biodiversity and specific ecosystem services at different spatial scales, from local to global, these studies typically used taxonomic diversity as a measure of biodiversity^15^. To this end, a recent meta-analysis found a spatial covariance between biodiversity, largely intended as species richness, and ecosystem service supply, such as carbon storage, water purification and crop pollination^15^. Recent evidence suggests that global mismatches in distribution patterns of mammals between taxonomic, phylogenetic and functional diversity exist, and may be largely driven by environmental conditions, such as mean annual temperature^16^. The biome level representation analysis presented here seems to align with the above mentioned pattern. This analysis (see results in Fig. 3) shows, for example, that deserts and xeric shrublands are better represented in the top priority areas when these are identified based on phylogenetic rather than taxonomic or functional diversity. Similar discrepancies between global priority areas for bird conservation identified based on species richness or evolutionary metrics have been reported^17^. Our results indicate that priorities identified by considering all three components of biodiversity are more widely scattered along the latitudes as compared to priorities based on species richness only again in line with findings from those previous studies mentioned above. However, we here took a step forward from previous studies by relating the three biodiversity components with selected ecosystem services to show that global synergies and trade-offs exist.

Our results demonstrate that, if the Aichi Target 11 could be met, 17% of the terrestrial land could be optimally allocated to achieve high coverage (i.e. around or above 30%) of the global priorities for biodiversity and ecosystem services. Few recent studies have highlighted the potential for achieving the Aichi target 11 relative to global biodiversity protection^18–20^. Conversely, the potential synergies and trade-offs that these global protection strategies entail for the protection of ecosystem services have been often overlooked but are equally important. Specifically, here we identify potential synergies between different facets of diversity and carbon. The link between biodiversity, intended as species richness, and carbon conservation has been investigated, and synergies resulting from an integrated approach considering both biodiversity and carbon have been previously identified^5^. Here we show that similar synergies between biodiversity and carbon emerge also when the different facets of biodiversity, such as phylogenetic and functional diversity, in addition to taxonomic diversity, are jointly considered. This result overall reinforces the assertion that there may be significant opportunities for capitalizing on carbon sequestration strategies, e.g. through international schemes such as the initiative for reducing emissions from deforestation and forest degradation (REDD+)^21^. This would simultaneously aid the protection of areas not only rich in species numbers, but also areas of high importance where species of unique evolutionary history and ecological functions occur. Together, climate change and the biodiversity collapse represent two of the main environmental challenges of our times^5^. Carbon conservation would not only help to mitigate climate change but, as we report here, would also aid broader conservation of biodiversity and other ecosystem services.

The synergies we found were not restricted to biodiversity and carbon only, but also extend to the supply of freshwater recharge, a result that is in line with the findings of a study showing large synergies between biodiversity, carbon storage and freshwater services^4^. This latter synergy may have particularly relevant implications in a changing world. Water is broadly regarded as an essential natural resource for human societies, yet it is highly threatened by anthropogenic activities, including climate change^22^. Thus, our finding that an integrated approach considering ecosystem services and biodiversity identified synergies between biodiversity, carbon and also water supply services is encouraging towards the development of effective policies aimed at achieving biodiversity conservation and ecosystem services globally. Such policies, e.g. payments for ecosystem services, not only have the potential to avert the widespread decline of biodiversity across its multiple facets, but can also aid climate mitigation and water preservation, which are essential in supporting the current and future wellbeing of human societies.

However, our findings also highlight important trade-offs. Specifically, we show that when priority areas are identified considering biodiversity only, ecosystem services, such as pollination, would potentially remain largely unprotected. This, finding, while relevant at the global scale, underscores the importance of considering both biodiversity and selected ecosystem services when seeking to prioritize areas for both these essential components, because an exclusive focus on biodiversity may prove ineffective in protecting some ecosystem services. However, we also show that using an integrative approach that combines the different components of biodiversity as well as a selection of ecosystem services appears to yield an optimal and most balanced solution. This holistic approach not only allows a very similar coverage of the biodiversity components as that achieved when prioritizing for biodiversity only, but also allows high coverage of ecosystem services, such as pollination. These latter services would have otherwise been largely unprotected in a prioritization exclusively focused on biodiversity.

As with previous global conservation planning assessments a number of caveats need to be highlighted in this study. The present analysis was carried using data at a resolution of 110 × 110 km and based on species occurence maps susceptible to comission errors (when a species is mistakenly thought to be present) and omission errors (when a species is mistakenly thought to be absent). In the case of this study, given the very coarse resolution of the analyses, biases related to commission and particularly omission, errors are deemed to be minimal. Overall, the results presented here are valuable in highlighting global patterns, and are largely based on biodiversity metrics that represent the potential, rather than the real diversity of species in a given landscape unit. Similar analyses done at the national or regional scale should take into account species distribution data at finer resolution or redefine species ranges on the basis of the habitat requirements of the species. Local and regional analyses would be particularly relevant for this type of studies, so as to gather scientific evidence of the covariation of biodiversity and ecosystem services at a scale that would match that at which management efforts are typically implemented^15^. Moreover, here we used the same coarse resolution across all the spatial layers of information available. This may introduce uncertainty and issues of scale relative to the spatial extent that is relevant to e.g. each single ecosystem service considered. For example, pollination services may be best captured by local scale patterns of landscape compositions and configuration that were not necessarily represented in our data^23^.

Ultimately, as stated above, the results of this study are intended to uncover global patterns of covariance among biodiversity and selected ecosystem services. They may pave the way to more in depth investigations on the links between these components at the level of biomes, regions or beyond. In addition, our study was restricted to a small selection of ecosystem services for which global spatial layers were available. We acknowledge that considering other services would have been relevant, but somewhat difficult to achieve given current limitations in the availability of spatial data. The ecosystem services considered here are also largely referring to supply services rather than values. This is especially true for services such as water recharge, which may largely locate in remote areas where they may not be of direct value to humans. Moreover, our identified priority areas for biodiversity and ecosystem services partly overlap with areas of high value for current and future food production^24^. Therefore, future studies should attempt to incorporate food production potential as an additional and crucially important component in order to expand our understanding on trade-offs and synergies for conserving biodiversity, preserving ecosystem services and at the same time feeding the world.

Overall, we show that synergies between important areas for ecosystem services and biodiversity conservation exist, and that there is scope for aligning priorities for the multiple facets of biodiversity and ecosystem services. While the present findings carry implications for global scale conservation policies, regional to local conservation planning exercises should be carried out within the identified global priority areas in order to guide implementation of effective management efforts. Ultimately, our results from the integrated scenario call for a shift towards integrative and holistic approaches that explicitly incorporate not only the biodiversity dimensions but also other fundamental components, such as ecosystem services, when seeking to identify important areas for conservation.

## Methods

### Species distribution data

Distribution data for mammals were obtained from the IUCN^25^, while data for birds were obtained from Birdlife International^26^. The data sets were available as geographic information system (GIS) polygons, covering known or inferred areas of occurrence of the species. The polygons were converted to rasters on an equal-area Molleweide projection, with a resolution of 1° equivalent (110 × 110 km). Strictly marine mammal species and seabirds were excluded from the analyses.

### Measuring taxonomic (TD), functional (FD) and phylogenetic diversity (PD)

TD was derived by calculating the sum of the species occuring within each grid cell. FD and PD were calculated as follows: Trait data were derived from a comprehensive new database, containing species-level trait data for the world’s bird and mammal species^27^. The traits used in the analyses (a total of 15 for mammals and 19 for birds) comprised body mass, diet, habit and activity period (Table S1). A functional tree was built with the trait variables using hierachical clustering. Hierarchical clustering algorithms create a series of nested clusters, where the members of inferior-ranking clusters become members of larger, higher-ranking clusters. We performed the clustering using the UPGMA algorithm^28^. Body mass was log-transformed prior to all analyses. We used recently published phylogenies for mammals^29^ and birds^30^ PD was computed using the widely known metric known as Faith’s PD^31^. In brief PD is described as the sum of the branch lengths of a phylogenetic tree connecting all the species of a given assemblage^31^. Functional diversity was computed using the metric proposed by^32^ because it exactly parallels PD. Issues associated with comparing PD or FD values across assemblages can generally be summarized as being the result of decrease in the range of possible values as taxonomic diversity (species richness) of a community or assemblage approaches the number of species in the phylogeny or trait dataset. To permit comparative analyses, this artifact must be removed by quantifying whether the PD or FD is higher or lower than expected given the observed SR in the community or assemblage. We computed standardized effect sizes for both phylogenetic and functional diversity. Standardized effect sizes were calculated using fast algorithms based on exact expressions^33^.

### Ecosystem service data

Ecosystem service data included spatially explicit datasets on three ecosystem services: carbon biomass, groundwater recharge and pollination benefits. The carbon biomass data were obtained from^34^. This dataset consists of global estimates of above- and below-ground (root) carbon biomass densities (T/ha), including soil carbon. Carbon densities were mapped using the IPCC tier 1 values of vegetation biomass and a global land cover map stratified by continent, ecoregion, and forest disturbance level. Groundwater recharge data were obtained from^35^. Groundwater recharge, the amount of water that filters from the earth’s surface to replenish groundwater supplies, is an important ecosystem service. Since groundwater is protected from surface pollution and experiences less fluctuation in levels than surface water sources, lack of groundwater recharge can limit sustainable groundwater usage. Areas with high groundwater recharge are considered to be of high value. The dataset consists of long term averages (1961–1990) in groundwater recharge produced as part of the ouput of the WaterGAP hydrological model. In brief WaterGap computes time-series of fast-surface and subsurface runoff, groundwater recharge and river discharge as well as storage variations of water in canopy, snow, soil, groundwater, lakes, wetlands and rivers. Model input includes time series of climate data (e.g. precipitation, temperature and solar radiation) and physiogeographic information like characteristics of surface water bodies (lakes, reservoirs and wetlands), land cover, soil type, topography and irrigated area^36^. Groundwater recharge expressed in mm/y were calculated by aggregating the long-term average values from resolution of 0.5 × 0.5 to the resolution of all the other layers. The pollination benefits data were derived from^37^. Pollination benefits expressed in US$ per hectare were calculated by integrating information on crop distribution for 60 pollination dependent crops, product of producer price, production quantity and the extent of pollination dependency for each crop type.

### Selecting priority regions for biodiversity and ecosystem services

We used the spatial prioritization method Zonation to identify priority areas for ecosystem services and biodiversity^38^. Zonation identifies areas of high conservation priority by iteratively removing sites from the full landscape that contribute least to conservation targets, while taking othe specified cost factors into consideration. We used the additive benefit function of Zonation^38^. The additive benefit function bases selection on a cell’s weighted summed occurrence value over all features, thus changing the balance of selection to favor feature-rich areas over areas with a high occurrence value for one or a few species^38^. Six different conservation scenarios were produced, three in which each individual component of biodiversity drove the prioritization, one combined scenario for all biodiversity components, one scenario for ecosystem services alone and one combining all three biodiversity components together with ecosystem services. In Zonation analyses spatial layers can be assigned different weights so that some features are prioritized over others. In our analyses all features (i.e. each of the three ecosystem service layers) were weighted equally, so that the weight of a single ecosystem service feature was equal to the aggregate weight of the biodiversity layers. In practice when considering the individual components of biodiversity we assigned each biodiversity component a weight of 1. In the combined scenario we assigned to each biodiversity feature a weight of 1, so that the total sum of the weights for all biodiversity components would be 6. We assigned each ecosystem service a weight of 6. The output of the zonation analyses describes the extent to which each feature was retained in any given high- or low-priority fraction of the landscape.

### Sensitivity analyses

We performed a sensitivity analysis aimed a testing the effects of individual features (e.g. each of the biodiversity components or each of the three ecosystem services) on the results of the prioritization exercise. In doing so, we progressively increased the weights of a given feature, while keeping constant the weights of all the features. We then in turn calculated the proportion of each feature contained within the top 17% fraction of the ranked landscape as the weight of one feature at a time was changed. This exercise allowed us to verify the extent to which changes in weight of one feature affect the prioritization outcome for this and for the other features.

## Supplementary information

Supplementary Information
