# Supplementary material for: Global synergies and trade-offs between multiple dimensions of biodiversity and ecosystem services
Source: Sci Rep. 2019 Apr 4;9:5636. doi: 10.1038/s41598-019-41342-7 (PMC6449357; doi:10.1038/s41598-019-41342-7)
Supplement: Supplementary file 1 — Supplementary Information [file 41598_2019_41342_MOESM1_ESM.pdf]

Supporting Information

Global synergies and trade-offs between multiple dimensions of biodiversity and ecosystem services

Marco Girardello, Andrea Santangeli, Emiliano Mori, Anna Chapman, Simone Fattorini, Robin Naidoo, Sandro Bertolino, Jens-Christian Svenning

Table S1 Traits used to measure mammal and bird functional diversity. These traits represent many aspects of resources use.See methods for further details on how these traits have been used and the original source of these trait data.

**Trait type**	**Trait**	**Scale and categories**
Resource quantity	1. Body mass	Continuous
Main components of diet(s)	2. Percent use of: invertebrates	estimated % use
	3. Percent use of: mammals and birds	estimated % use
	4. Percent use of: reptiles	estimated % use
	5. Percent use of: fish	estimated % use
	6. Percent use of: vertebrates-general or unknown	estimated % use
	7. Percent use of: scavenge, garbage, offal, carcasses, trawlers, carrion	estimated % use
	8. Percent use of: fruit, drupes	estimated % use
	9. Percent use of: nectar, pollen, plant exudates, gums	estimated % use
	10. Percent use of: seed, maize, nuts, spores, wheat, grains	estimated % use
	11. Percent use of: other plant material	estimated % use
Main foraging method(s)	12. M - marine, G - ground level including aquatic foraging (see ForStrat-Comment) S- scansorial, Ar - arboreal, A- aerial.	categorical
Main foraging period (s)	13. Activity-Nocturnal	categorical (binary)
	14. Activity-Crepuscular	categorical (binary)
	15. Foraging activity at day	categorical (binary)

Table S2 Correlation among biodiversity facets and ecosystem services (spearman correlation coefficients). Values represent correlation coefficients obtained from pixel-level values of biodiversity ecosystem service metrics.

	TD mammals	FD mammals	PD mammals	TD birds	FD birds	PD birds	Water	Carbon	Livestock	Pollination
TD mammals	1									
FD mammals	0.32	1								
PD mammals	0.38	0.26	1							
TD birds	0.87	0.23	0.34	1						
FD birds	0.58	0.06	0.46	0.73	1					
PD birds	0.48	0.06	0.6	0.51	0.74	1				
Water	0.12	-0.08	0.11	0.03	0	0.03	1			
Carbon	0.66	0.19	0.38	0.68	0.55	0.39	0.05	1		
Livestock	0.36	-0.12	0.17	0.37	0.29	0.27	0.32	0.17	1	
Pollination	0.25	-0.04	0.15	0.2	0.17	0.15	0.59	0.23	0.34	1

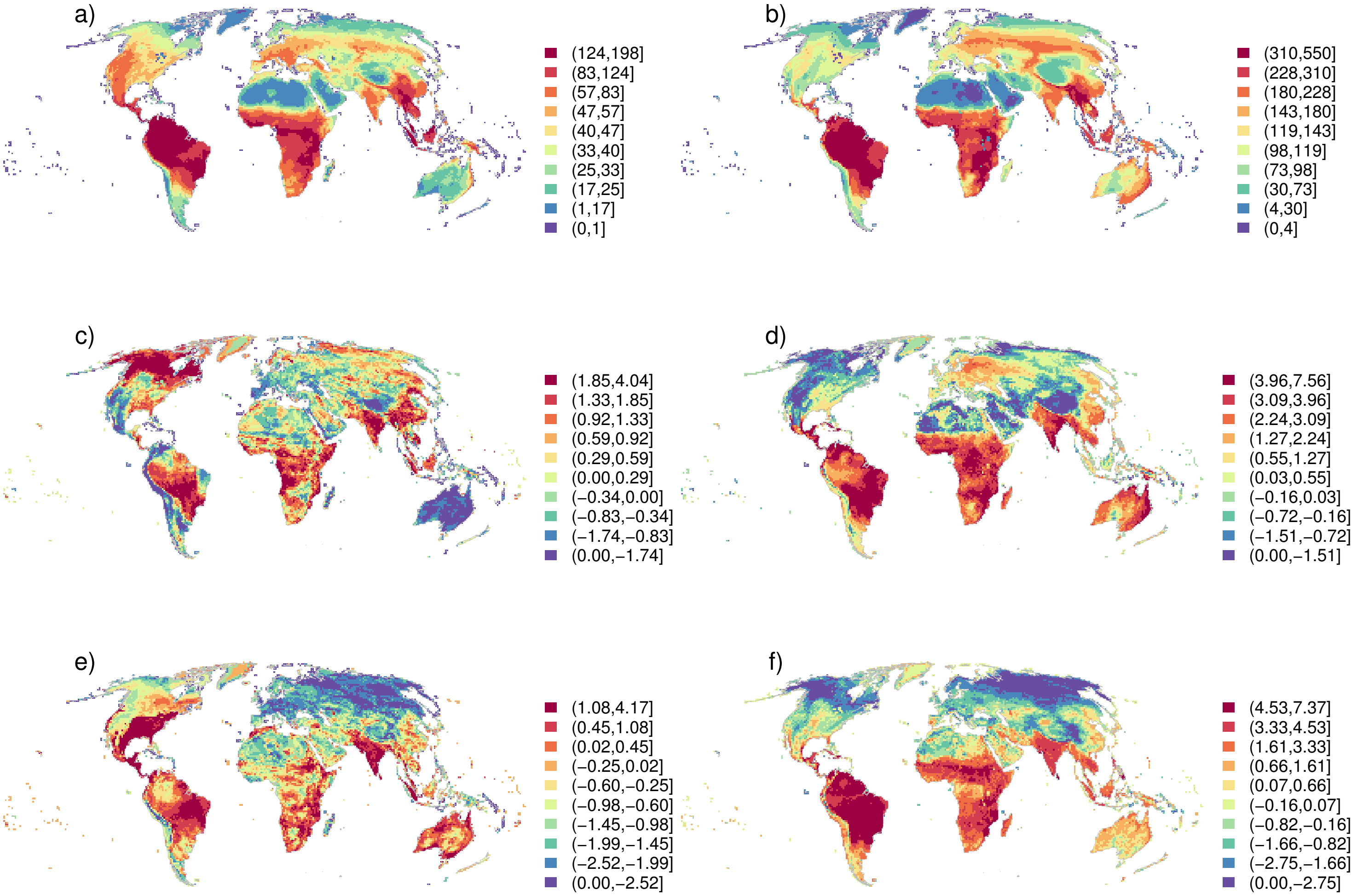

Figure S1 Global maps of taxonomic, functional and phylogenetic diversity for birds and mammals. a) taxonomic diversity-mammals b) taxonomic diversity-birds c) functional diversity-mammals d) functional diversity- birds e) phylogenetic diversity-mammals f) phylogenetic diversity-birds. Taxonomic diversity was calculated as the sum of the species for each grid cell. Phylogenetic diversity and functional diversity were calculated as the sum of the branch lengths of a phylogenetic functional/tree connecting all the species of a given assemblage. Phylogenetic and functional were standardized for species richness using the procedures described in Tsirogiannis & Sandel (2016).

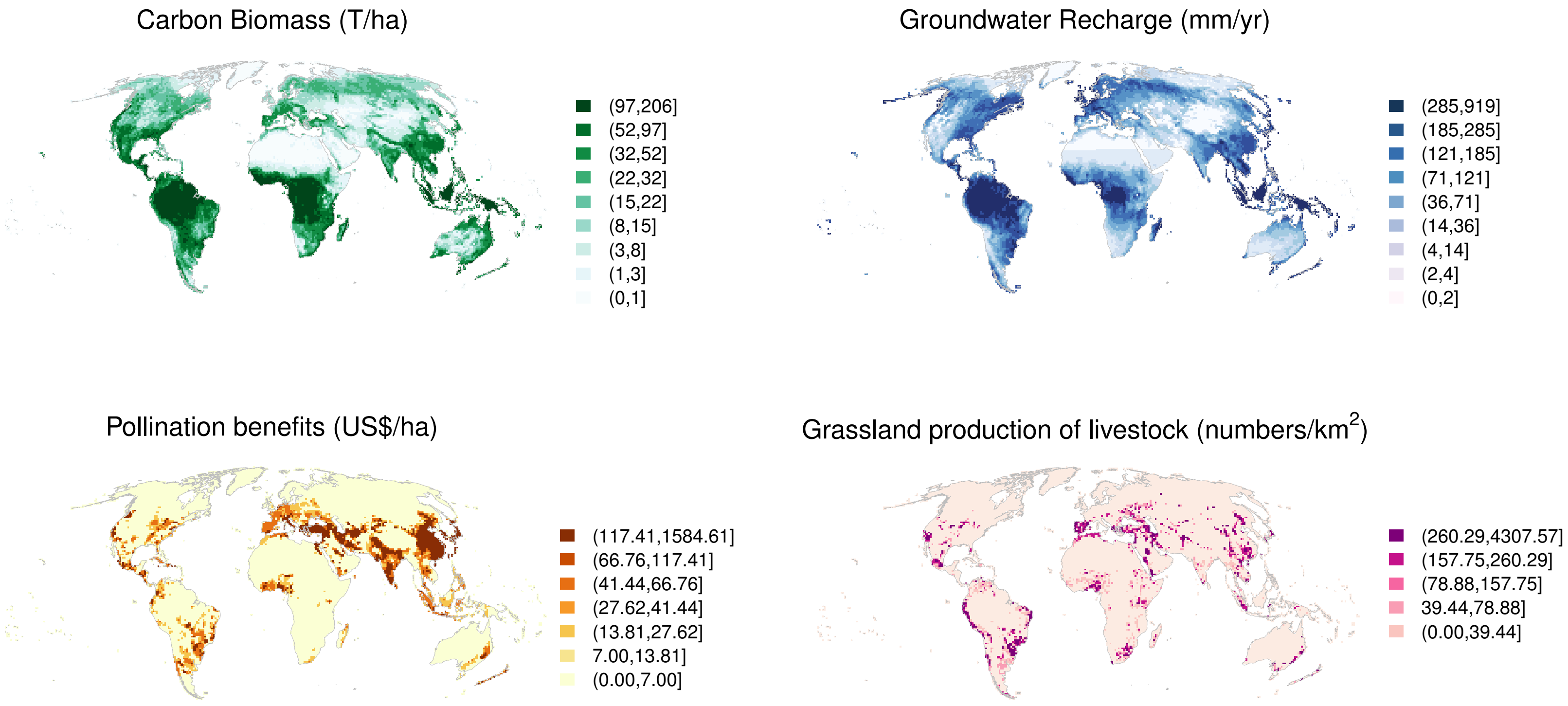

Figure S2 Ecosystem service maps used in the prioritization analysis. All the ecosystem service data were resampled to a resolution of 110x110km i.e. the same resolution of the biodiversity data. A detailed description of the sources of ecosystem service data are given the methods section of the main text.

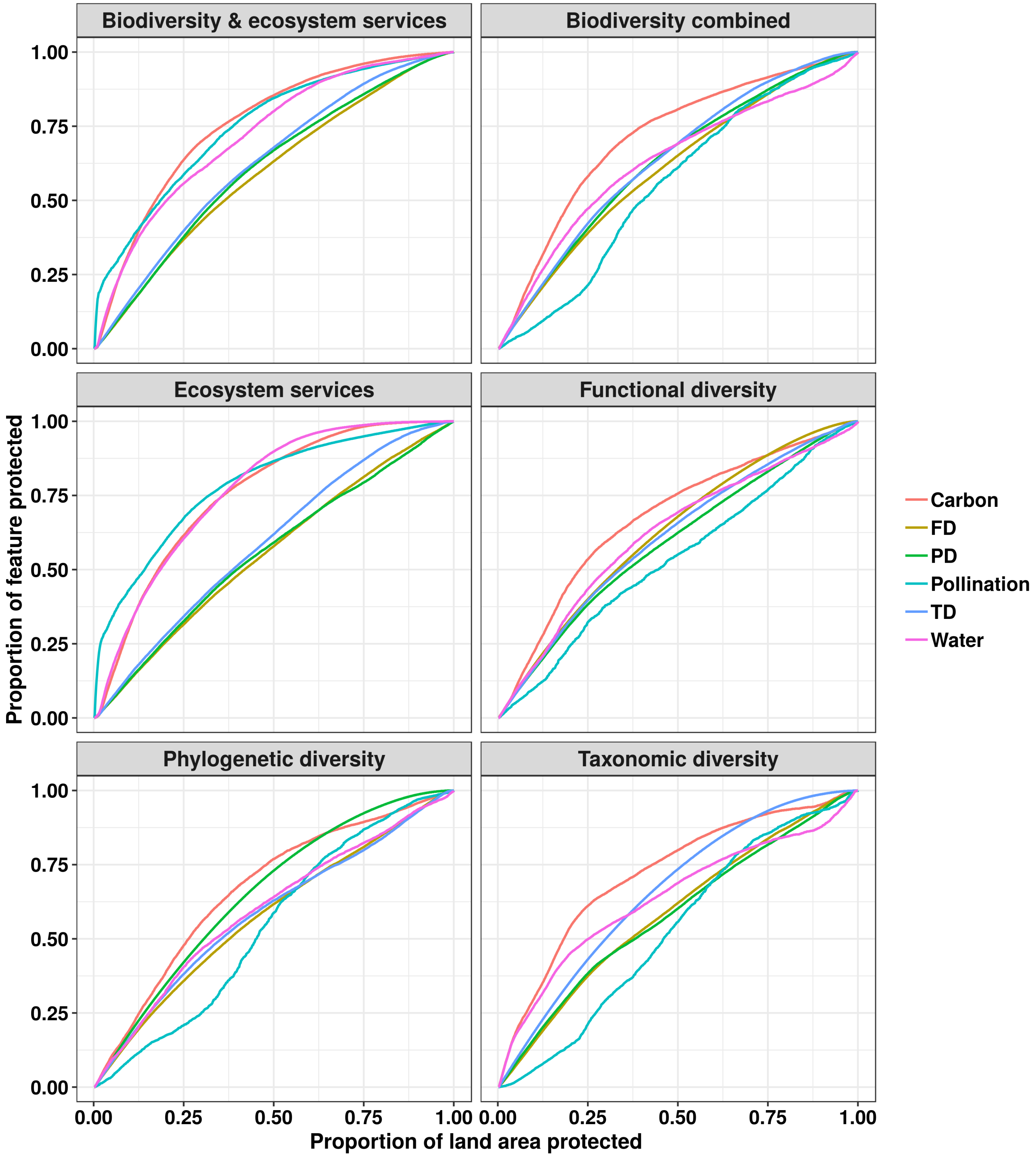

Figure S3 Proportion of each feature retained (Y axis: from 0, i.e. the feature is left completely unprotected, to 1, the feature is totally protected across its distribution.) in each conservation solution as a function of the the land area protected ranging from 0, i.e. the whole study landscape is virtually unprotected, to 1, i.e. all of the study landscape is virtually protected. The curves depict trade-offs between different features here represented by a line of a specific color during the landscape ranking process. Each feature (biodiversity component or ecosystem service) is depicted with a different colour.

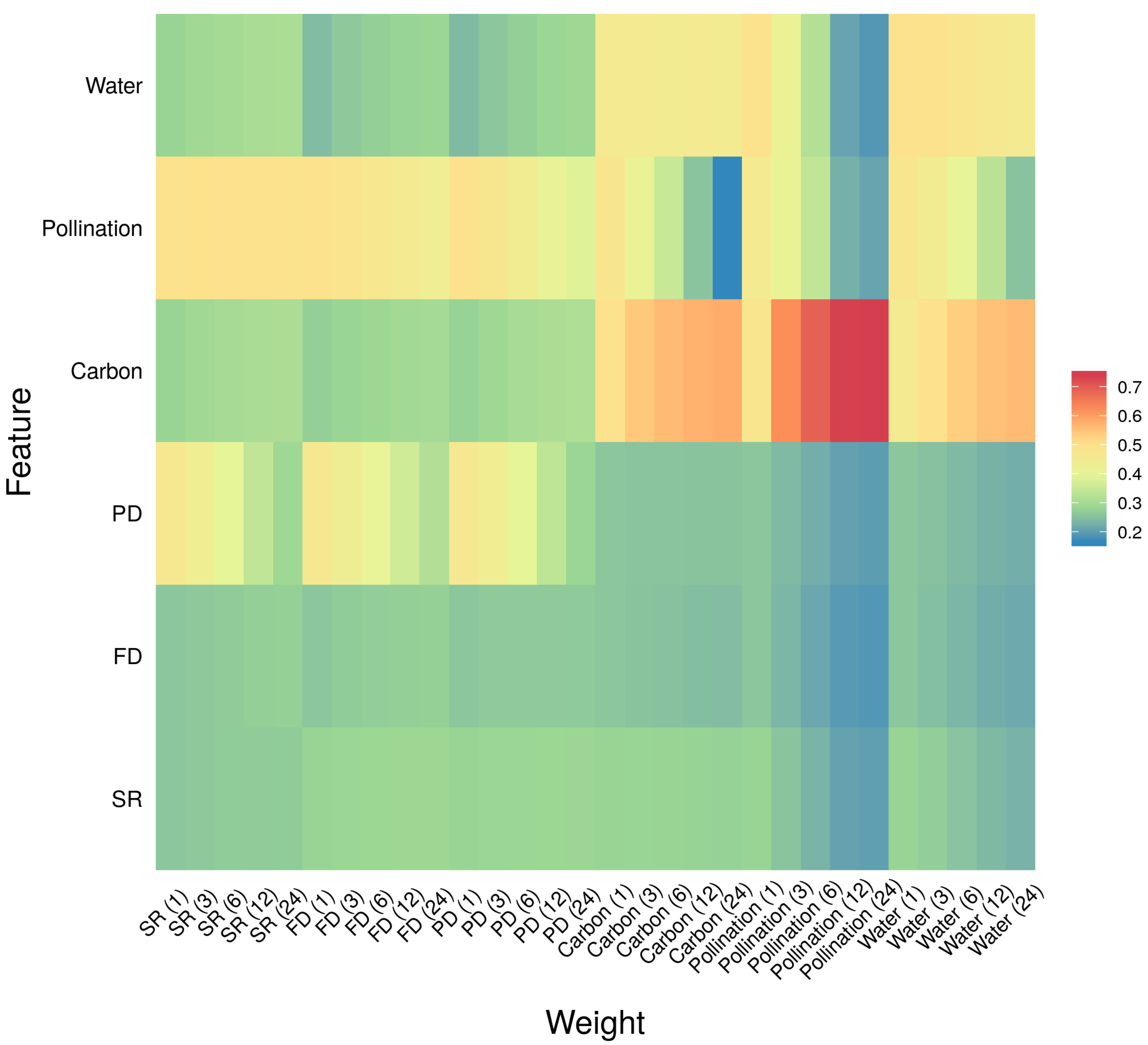

Figure S4 The proportion of each feature retained in the top 17% fraction of the landscape when the weight of a given feature was varied, while keeping the weights for all the other features constant. The red colour indicates that a higher proportion a particular feature was retained in the final lanscape.

References

Tsirogiannis, C. & Sandel, B. (2016). PhyloMeasures: A package for computing phylogenetic biodiversity measures and their statistical moments. Ecography, 39(7), 709-714.
